# An Amplifier-Less Acquisition Chain for Power Measurements in Series Resonant Inverters

**DOI:** 10.3390/s19194343

**Published:** 2019-10-08

**Authors:** Jorge Villa, José I. Artigas, Luis A. Barragán, Denis Navarro

**Affiliations:** Department of Electronic Engineering and Communications, I3A, Universidad de Zaragoza, María de Luna 1, 50018 Zaragoza, Spain; jvillal@unizar.es (J.V.); barragan@unizar.es (L.A.B.); denis@unizar.es (D.N.)

**Keywords:** analog-digital conversion, data acquisition chain, error compensation, power measurement, home appliances, induction heating

## Abstract

Successive approximation register (SAR) analog-to-digital converter (ADC) manufacturers recommend the use of a driver amplifier to achieve the best performance. When a driver amplifier is not used, the conversion speed is severely penalized because of the need to meet the settling time constraint. This paper proposes a simple digital correction method to raise the performance (conversion speed and/or accuracy) when the acquisition chain lacks a driver amplifier. It is intended to reduce the cost, size and power consumption of the conditioning circuit while maintaining acceptable performance. The method is applied to the measurement of the output power delivered by a series resonant inverter for domestic induction heating.

## 1. Introduction

**Figure 1:**
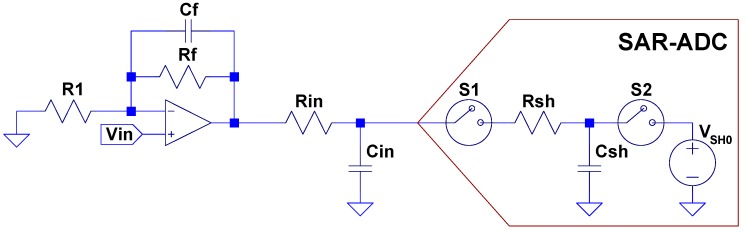
Successive approximation register (SAR) analog-to-digital converter (ADC) input driving circuit recommended by manufacturers.

Successive approximation register (SAR) analog-to-digital converter (ADC) manufacturers recommend the use of a front-end circuit that consists of two parts: a driving amplifier and an RC filter [1,2]. The former can be used to implement a low pass anti-aliasing filter as shown in Figure 1; the $R_{in}$, $C_{in}$ components are needed to isolate the driving circuit from the kick due to the switched capacitors of the ADC’s input structure. Selection of the amplifier and $R_{in}$, $C_{in}$ values are critical to achieving the best performance [3].

The sensor can be connected directly to the SAR-ADC without any input driver amplifier in applications where the input signal bandwidth is much smaller than the sampling frequency. However, this low-cost solution requires the input signal to settle to within 1/2 of the least significant bit (LSB) to maintaining the conversion accuracy [4]. ADC’s acquisition time increases, and this dramatically reduces the throughput rate. Removing the input driver amplifier can have other advantages besides lowering the cost. The designer is no longer concerned with the noise or distortion introduced by the driver amplifier itself. Another potential problem avoided is the dynamic requirements that are placed on the amplifier by the ADC, and the stability issues inherent to any closed-loop structure.

Several error compensation methods for ADC are reviewed in [5]. Track and hold non-linearity of a high-performance ADC is modeled in [6], and a digital algorithm is proposed to reduce the distortion. Recent advances in methods to raise the performance of SAR-ADC are summarized in [7]. Nevertheless, none of the works focus on the errors resulting from removing the driver amplifier in the acquisition chain.

This paper proposes a simple digital correction method to get over the throughput limitation due to the settling time when driving the ADC without any amplifier. The method is based on the approximation of the sample and hold (SH) circuit by a first-order RC network. The correction is performed by only one addition and multiplication per sample.

**Figure 2:**
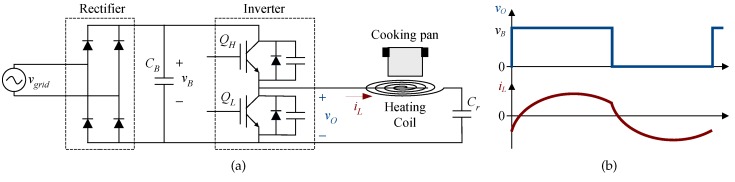
Domestic induction heating power converter: (**a**) Schematic. (**b**) Output waveforms.

In addition to low-demanding consumer applications, this innovative solution can be utilized in medium-throughput systems. For instance, in this paper, it is applied to the power measurement of a domestic induction heating (DIH) appliance, where low-cost implementation is required [8]. Figure 2a shows the schematic of the power converter considered. It is based on a half-bridge series resonant inverter (HBSRI). The bus voltage ($v_{B}$) is a full-wave rectified mains waveform filtered with a bus capacitor ($C_{B}$) designed to allow a big ripple. The power delivered to the load is controlled by varying the operating switching frequency ($f_{sw}$) usually in the range of 30–75 kHz [9].

Several methods have been reported to measure the output power for DIH. The most accurate method is to acquire the load voltage drop and the current that flows through the inductor by two ADCs with a high enough sampling rate and resolution. However, given the low-cost context of DIH applications, and assuming the equivalent series resistance (ESR) of the resonant capacitor is negligible, a similar accuracy in the computation of the power can be achieved by acquiring only the inverter output voltage ($v_{O}$) and the inductor current ($i_{L}$) (Figure 2). Since DIH is a cost-oriented application, first-order and second-order single-bit sigma-delta ADCs have also been reported [10,11].

In this work, two SAR-ADCs in the range of several MSPS are used to acquire the inverter output voltage and the inductor current. The digital correction method is implemented in a system-on-chip (SoC), whose architecture integrates the processing system (PS)—based on a dual-core ARM Cortex-A9 processor—and the programmable logic (PL), which consists of field-programmable gate array (FPGA) fabric [12]. These two parts communicate to each other through the Advanced eXtensible Interface (AXI) 4. Although an SoC is used for the validation of the proposal, given the low-cost framework of DIH applications, the final implementation of the proposed algorithms would target the application-specific integrated circuit (ASIC) or the microcontroller already available for control and protection purposes of the power converter.

This paper is organized as follows. Section 2 develops the correction method and describes the laboratory setup utilized in this work. Section 3 reports the experimental results. Finally, Section 4 summarizes the main conclusions.

## 2. Materials and Methods

### 2.1. SAR-ADC Acquisition Chain Analysis

The structure of an SAR-ADC includes an SH circuit to track and hold the changing input signal, a digital-to-analog converter (DAC) that converts the estimated digital value to an analog value, a comparator and a SAR circuit that obtains the digital output by comparing the sampled input signal with the DAC output voltage [7]. The most standard structure for the DAC is based on a binary-weighted capacitor array. During conversion, the charge of the SH capacitor is redistributed through the capacitor array.

**Figure 3:**
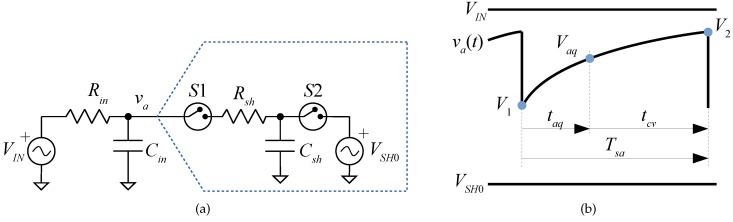
Circuit analysis: (**a**) front-end and sample and hold (SH) schematic, and (**b**) waveforms.

Figure 3a shows the input front-end without amplifier connected to an SAR-ADC. $V_{IN}$ represents the input voltage to be converted, $R_{in}$ is the equivalent input resistance connected to the ADC, and $C_{in}$ is the equivalent input capacitance resulting from a charge-reservoir capacitor and the parasitic capacitance of the ADC input pin. These passive components also play the role of a low pass anti-aliasing filter.

Figure 3a also shows a simple equivalent input circuit for the ADC [3], where $C_{sh}$ represents the SH capacitor, $R_{sh}$ is the input equivalent resistance, S1 and S2 model the sampling and conversion switches, and $V_{SH0}$ is a DC voltage source whose value depends on the charge redistribution circuit. Depending on the converter’s input structure, this voltage can be equal to the input during the previous conversion, ground, or the reference voltage $V_{REF}$ of the ADC [3]. Newer ADCs can have a voltage of $V_{REF}/2$ or another fraction of $V_{REF}$.

The circuit will be analyzed for the case $R_{sh}<<R_{in}$ and $V_{IN}$ a DC constant value. Figure 3b shows the resulting voltage waveforms during the ADC operation. The sampling period $T_{sa}$ of the ADC can be split into two parts: the acquisition time $t_{aq}$ (S1 on, S2 off) and the conversion time $t_{cv}$ (S1 off, S2 on).

The input voltage $V_{IN}$ results in an actual sampled voltage $V_{aq}$. The proposed correction method will attempt to recover the input voltage from the actual sampled voltage. The following time constants will be used to find the low pass anti-aliasing filter relationship between the variables of interest:

(1) $$\tau_{in}=R_{in}C_{in}$$

(2) $$\tau_{sh}=R_{in}C_{sh}$$

(3) $$\tau_{aq}=\tau_{in}+\tau_{sh}$$

Just before the acquisition time, $C_{in}$ is charged at a voltage $V_{2}$ and $C_{sh}$ at $V_{SH0}$. When S2 opens and S1 closes both capacitor voltages become equal to $V_{1}$ as the charge quickly redistributes between $C_{in}$ and $C_{sh}$ through the small $R_{sh}$ resistor (falling edge of $v_{a}\left(t\right)$ in Figure 3b). The charge redistribution follows the equation:

(4) $$V_{1}=\frac{\tau_{in}}{\tau_{in}+\tau_{sh}}V_{2}+\frac{\tau_{sh}}{\tau_{in}+\tau_{sh}}V_{SH0}.$$

During the acquisition time—S1 closed and S2 open—the ADC input voltage $v_{a}\left(t\right)$ can be approximated by the step response of a first-order differential equation with a time constant $\tau_{aq}$. The relationship between $V_{aq}$ and $t_{aq}$ is given by:

(5) $$V_{IN}-V_{aq}=\left(V_{IN}-V_{1}\right)e^{-t_{aq}/\tau_{aq}}.$$

During the conversion time—S1 open and S2 closed—the ADC input voltage $v_{a}\left(t\right)$ is given by a first-order differential equation with a time constant $\tau_{in}$. Solving the differential equation during the conversion time yields:

(6) $$V_{IN}-V_{2}=\left(V_{IN}-V_{aq}\right)e^{-t_{cv}/\tau_{in}}.$$

By solving $V_{aq}$ as a function of $V_{IN}$ in the system of equations given by (4)–(6) we get:

(7) $$V_{aq}=\left(1-\alpha \right)V_{IN}+\alpha V_{SH0}$$

where:

(8) $$\alpha =\frac{e^{-t_{aq}/\tau_{aq}}}{1+\frac{\tau_{in}}{\tau_{sh}}\left(1-e^{-t_{aq}/\tau_{aq}-t_{cv}/\tau_{in}}\right)}.$$

Equation (7) will be used in the next subsections for calibration purposes.

### 2.2. Correction Method

The voltage $V_{aq}$ is the actual sampled voltage by the ADC because $C_{sh}$ tracks the ADC input voltage $v_{a}\left(t\right)$ during the acquisition time. The difference between $V_{IN}$ and $V_{aq}$ is the conversion error to be corrected. One way to decrease this error is to increase the acquisition time, as explained in [4]. The other way is the proposed in this work: To digitally recover $V_{IN}$ from $V_{aq}$ allowing for higher throughput. By solving for $V_{IN}$ in (7), we get the following correction equation:

(9) $$V_{IN}=k_{1}V_{aq}-k_{2}$$

where:

(10) $$k_{1}=\frac{1}{1-\alpha }$$

(11) $$k_{2}=\frac{\alpha }{1-\alpha }V_{SH0}.$$

Equation (9) will be applied to the digital acquired data to decrease the acquisition error even when $t_{aq}$ is far below the settling time of 1/2 LSB.

Values for $k_{1}$ and $k_{2}$ depend on several timing parameters and voltage $V_{SH0}$. Times $t_{aq}$ and $t_{cv}$ are determined from the ADC control scheme. Time constants can be computed from the discrete RC components and the ADC data-sheet. $V_{SH0}$ can be obtained from the ADC data-sheet, or it can be guessed from the experimental measurement of the ADC input-voltage driven by a signal generator with high enough source resistance [3].

One problem with this method is that the dynamic range is narrowed because $v_{a}\left(t\right)$ can not expand over the full-scale range. This problem is minimized by selecting a value high enough for $C_{in}$ to reduce $v_{a}\left(t\right)$ ripple. Once the value for $C_{in}$ is selected, $R_{in}$ can be computed to set the required cut-off frequency of the anti-aliasing filter.

Another problem is that $k_{1}$ and $k_{2}$ depend on several time constants given by RC components. The actual values of these components will differ from their nominal values due to manufacturing tolerance, temperature dependence, and aging—especially for capacitors.

The correction method using nominal RC values will be denoted as “nom” in the experimental results section. Some calibration methods will be proposed in the next subsections to obtain more accurate values for the time constants as well as to correct possible bias errors. The calibration method depends on the conditioning circuit and the signal to be acquired. The next subsections address the problem for two cases of interest: the output voltage and the load current of the HBSRI inverter.

### 2.3. Voltage Acquisition

#### 2.3.1. Acquisition Circuit

**Figure 4:**
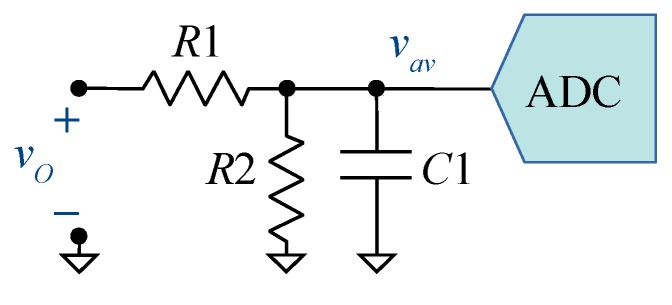
Acquisition chain for the inverter output voltage.

Figure 4 shows the acquisition chain used for the inverter output voltage $v_{O}$. It consists of a passive voltage divider, a filter capacitor, and an LTC2315-12 serial ADC from Linear Technology [13] with a reference voltage $V_{REF}=$ 3.3 V. The signal to be acquired is a variable frequency square voltage whose amplitude is given by the rectified bus voltage $v_{B}$ in Figure 2. The maximum amplitude is 340 V for a 240 V mains RMS value. The value of $C_{sh}=$ 10 pF is available in the data-sheet, but other parameters such as $R_{sh}$ and $V_{SH0}$ are missing. The value of $R_{sh}$ is not critical as long as it is far less than the equivalent source resistance R1||R2. The value for $V_{SH0}$—near to $V_{REF}/2$—has been obtained from the calibration process.

Resistors R1 and R2 have been calculated to allow a voltage range of 0–356 V. Capacitor C1 is selected to be much higher than $C_{sh}$ and to set a suitable cut-off frequency $f_{C}={\left(2\pi (R1||R2)C1\right)}^{-1}$. Table 1 shows the type and nominal values selected for the passive components. These values define a cut-off frequency $f_{C}=$ 525 kHz, high enough for several harmonics of $v_{O}$ to be acquired.

#### 2.3.2. Simple Calibration Method

Instead of using the component’s nominal values, a simple calibration method can be used for computing $k_{1}$ and $k_{2}$, to raise the accuracy of the correction method. It is based on performing one first acquisition with zero input voltage, i.e., by turning on the switch $Q_{L}$ of the HBSRI. When $V_{IN}$ is zero in (7), α can be computed exactly as:

(12) $$\alpha =\frac{V_{aq}}{V_{SH0}}.$$

From this value, $k_{1}$ and $k_{2}$ are computed with (10) and (11). In the experimental results section, this method will be denoted as “simple”. The online implementation of this method is straightforward.

#### 2.3.3. Optimization-Based Calibration Method

An extension of the above method consists in acquiring $v_{av}$ at several $t_{aq}$, with zero input voltage. Then, the acquired voltages are fitted to (7) by a non-linear optimization algorithm used to find the optimum time constants. Let $V_{avq0}$ be the vector of acquired voltages and $\left(p_{1},p_{2}\right)$ the parameters to find. Parameter $p_{1}$ is the factor that multiplies the nominal $\tau_{in}$ and $p_{2}$ is the factor that multiplies the nominal $\tau_{sh}$.

The optimization problem that must be solved is:

(13) $$\min_{p_{1},p_{2}}\sum_{k=1}^{NFIT}{\left(V_{avq}\left(p_{1},p_{2}\right)-V_{avq0}\right)}^{2},$$

subject to the following constraints:

(14) $$\begin{array}{c} 0.5\leq p_{1}\leq 1.5\\ 0.5\leq p_{2}\leq 1.5 \end{array}$$

where:

(15) $$V_{avq}\left(p_{1},p_{2}\right)=\left(1-\alpha \left(p_{1},p_{2}\right)\right)V_{IN}+\alpha \left(p_{1},p_{2}\right)V_{SH0},$$

and function $\alpha (p_{1},p_{2})$ derived from (8):

(16) $$\alpha \left(p_{1},p_{2}\right)=\frac{e^{-t_{aq}/\left(\tau_{in}p_{1}+\tau_{sh}p_{2}\right)}}{1+\frac{\tau_{in}p_{1}}{\tau_{sh}p_{2}}\left(1-e^{-t_{aq}/\left(\tau_{in}p_{1}+\tau_{sh}p_{2}\right)-t_{cv}/\left(\tau_{in}p_{1}\right)}\right)}.$$

In this paper we have used the *sqp*() function from Octave [14] which is an iterative method for constrained non-linear optimization.

When switch $Q_{L}$ is in the on state, $V_{IN}$ would ideally become zero. However, to account for possible bias offsets, the $V_{IN}$ value used in (15) will be computed as a suitable average of $v_{av}$ data acquired with high enough acquisition time. As $t_{aq}$ increases, the acquired data approaches asymptotically to $V_{IN}$.

**Figure 5:**
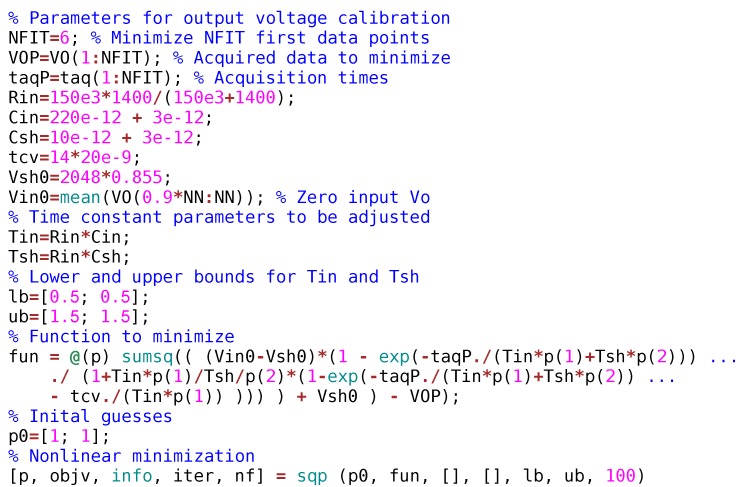
Octave code for the voltage calibration method.

Figure 5 shows the Octave code used to perform the optimization for voltage calibration. Variables VO and taq are vectors of length NN that contain the acquired values and their acquisition times, respectively. The acquisition time increases with the vector index. The first NFIT=6 data have been used for minimization. The last 10% data of VO are averaged to compute the Vin0 variable corresponding to $V_{IN}$ in (15). This code minimizes the objective function—denoted as fun()—by using a sequential quadratic programming (SQP) algorithm.

The correction constants $k_{1}$ and $k_{2}$ are computed with (10) and (11) by using the values found by optimization. In the experimental results section this method will be denoted as “opt”.

### 2.4. Current Acquisition

#### 2.4.1. Acquisition Circuit

**Figure 6:**
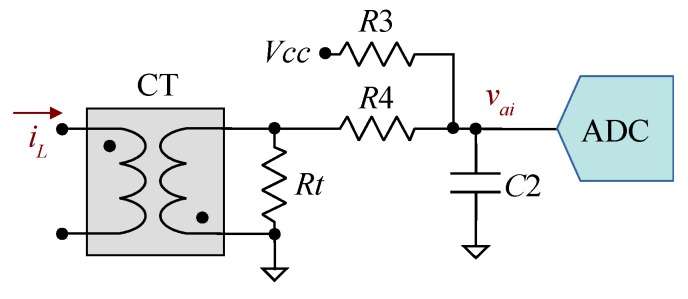
Acquisition chain for the inverter load current.

Figure 6 shows the acquisition chain used for the inverter load current $i_{L}$. It consists of a current transformer CT and a low-value resistor Rt to perform the I–V conversion, two resistors to add a DC level to the current signal, a filter capacitor and an LTC2315-12 serial ADC. A DC level has been added because the current is a symmetrical signal centered to zero, but the ADC has unipolar input.

Table 2 shows the type and nominal values selected for the passive components. The value of Rt allows a full-scale peak current of 80 A. Resistors R3 and R4 have the same value to add a dc value of Vcc/2. Vcc is the same voltage as the reference $V_{REF}$. Capacitor C2 is selected to define the same cut-off frequency as the output voltage circuit.

#### 2.4.2. Optimization-Based Calibration Method

In this case, it is not possible to apply the simple calibration method because a zero load current does not impose a zero equivalent $V_{IN}$. We will use an optimization-based method—the same as output voltage calibration—by acquiring $v_{ai}$ at several $t_{aq}$, with zero input current.

Let $V_{aiq0}$ be the vector of acquired voltages for zero input current. The optimization problem that must be solved is:

(17) $$\min_{p_{1},p_{2}}\sum_{k=1}^{NFIT}{\left(V_{aiq}\left(p_{1},p_{2}\right)-V_{aiq0}\right)}^{2}$$

subject to the same constraints as (14), where:

(18) $$V_{aiq}\left(p_{1},p_{2}\right)=\left(1-\alpha \left(p_{1},p_{2}\right)\right)V_{IN}+\alpha \left(p_{1},p_{2}\right)V_{SH0}$$

and function $\alpha (p_{1},p_{2})$ is the same as (16). With zero-load current, the equivalent $V_{IN}$ would ideally be Vcc/2. However, to have a more accurate value, we will use the same technique as for voltage calibration: The $V_{IN}$ value used in (18) will be computed as a suitable average of $v_{ai}$ values acquired with high enough acquisition time.

The correction constants $k_{1}$ and $k_{2}$ are computed with (10) and (11) by using the values found by optimization. In the experimental results section, this method will be denoted as “opt”.

### 2.5. Experimental Setup

**Figure 7:**
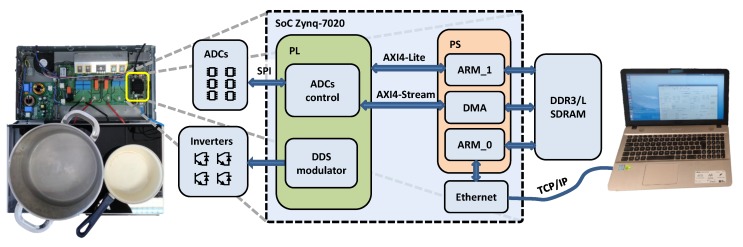
Experimental setup of the system.

The proposed correction and calibration methods have been tested on the experimental setup shown in Figure 7. It consists of a dual HBSRI fed by the rectified mains and controlled with a direct digital synthesis (DDS) modulator. The resonant inverters’ loads are two planar spiral inductors with external diameters of 21 and 15 cm. The high-diameter inductor has been used in this work. A commercial pot filled with water is over the inductor. The power converter is controlled by a Xilinx Zynq-7020 SoC running at a clock frequency of 100 MHz.

The prototype consists of four printed circuit boards (PCB): one big power converter board and three stacked controller boards. The top board of the stack is a commercial Trenz Electronic TE0720 [15]—an industrial-grade SoC module—integrating the Xilinx Zynq-7020 SoC and supporting circuitry: a gigabit Ethernet transceiver, 1 GB DDR3 (double data rate type 3) SDRAM (synchronous dynamic random-access memory) where data can be saved through direct memory access (DMA), 32 MB Flash memory for configuration, a USB transceiver, and switch-mode power supplies for all on-board voltages. A large number of configurable input/outputs (I/Os) is provided in a 4×5 cm module. The SoC module is mounted on the middle board—a Trenz Electronic TE0703 carrier board [16]—that provides low-cost connection and extension to the SoC module. It features a micro-SD card socket, USB connectors, and an RJ45 gigabit Ethernet socket. The carrier board is mounted on the bottom PCB, a custom acquisition board—designed for research and development purposes—that interfaces to the power converter board. This board includes the acquisition chains for several power converter signals.

The Zynq SoC generates the ADC serial peripheral interface (SPI) signals with a 50 MHz serial clock (SCK) frequency. The timing scheme of SCK held high during acquisition has been implemented to achieve the maximum throughput [13]. The sampling period $T_{sa}$ is the sum of 14 SCK conversion cycles—according to the LTC2315-12 data-sheet—and the acquisition time set by an integer number *n* of clock cycles. The resulting sampling rate, $f_{sa}$, is given by:

(19) $$f_{sa}=\frac{f_{SCK}}{14+n/2}.$$

For the case n=5, $f_{sa}=$ 3.03 MSPS. If we had to meet the 1/2 LSB settling time constraint, $t_{aq}$ would have been higher. Considering the voltage acquisition circuit (Figure 4) and a *B*-bit ADC [3,4]:

(20) $$t_{aq}\geq \left(R1||R2\right)\cdot C1\cdot ln\left(2^{B+1}\right).$$

Equation (20) yields a minimum $t_{aq}$ of 2.75 μs for a 12-bit ADC, and the sampling rate would have been reduced to only 0.33 MSPS.

To verify the proposed method, we have acquired the output voltage and load current of one HBSRI. The Chroma 61605 programmable AC power source has been used to feed the power converter, to get pure, instrument grade AC power. The reference data to compare with were collected using an oscilloscope in high-resolution acquisition-mode, with a sampling frequency of 100 MSPS. A high-voltage differential probe has been used to acquire voltage. Current acquisition was performed by inserting a precision current monitor in series with the load. We have used the Pearson current monitor model 411, with 20 MHz bandwidth and a sensitivity of 0.1 V/A +1/−0%.

## 3. Results

**Figure 8:**
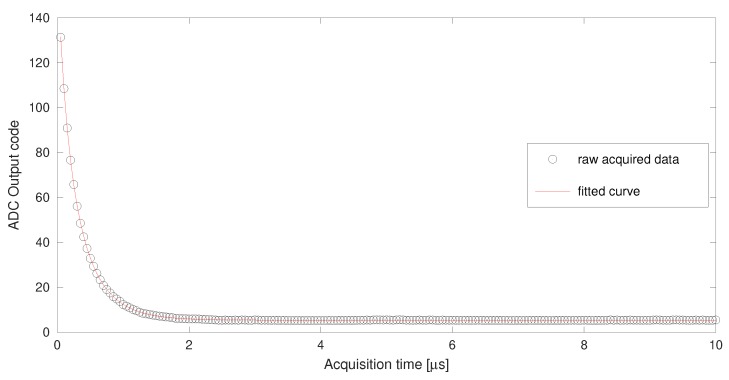
Acquired voltage fitting for zero output voltage.

Figure 8 shows the acquired voltage fitting for zero output voltage. The acquisition time spans 10 μs to reach a deep stationary state. The average of the last microsecond data is used as $V_{IN}$ in (18). The optimization process has been performed on the first six data with $t_{aq}$ in the range 50–300 ns with 50 ns steps. The minimization of the sum of squares of the differences (objv variable in Figure 5) results in a value of 0.3154. Such a low value explains the very good agreement of the data with the fitted curve.

**Figure 9:**
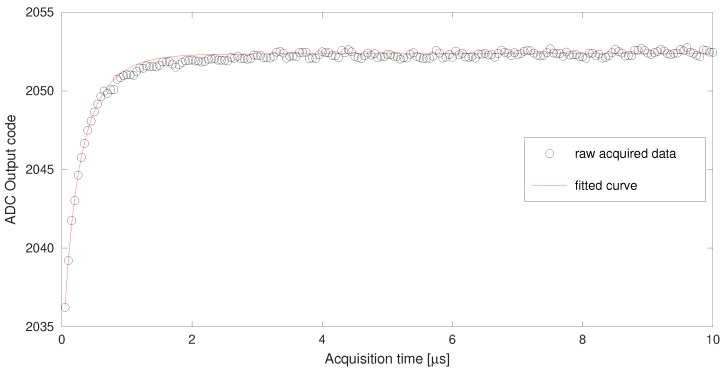
Acquired current fitting for zero load current.

Figure 9 shows the acquired current fitting for zero load current. The optimization process has also been performed on six data with $t_{aq}$ in the range 50–300 ns. The raw acquired data present an appreciable ripple due to the switched-mode voltage source that generates Vcc in Figure 6. However, the data are closely fitted to the curve, especially for low acquisition times.

The resulting values of the parameters found with the minimization process are summarized in Table 3.

Three magnitudes have been selected to verify the improvement achieved by the correction method: the RMS value of the output voltage ($V_{Orms}$), the RMS value of the load current ($I_{Lrms}$), and the average power delivered to the load (Power). All magnitudes have been computed over one half-cycle of the AC source.

**Figure 10:**
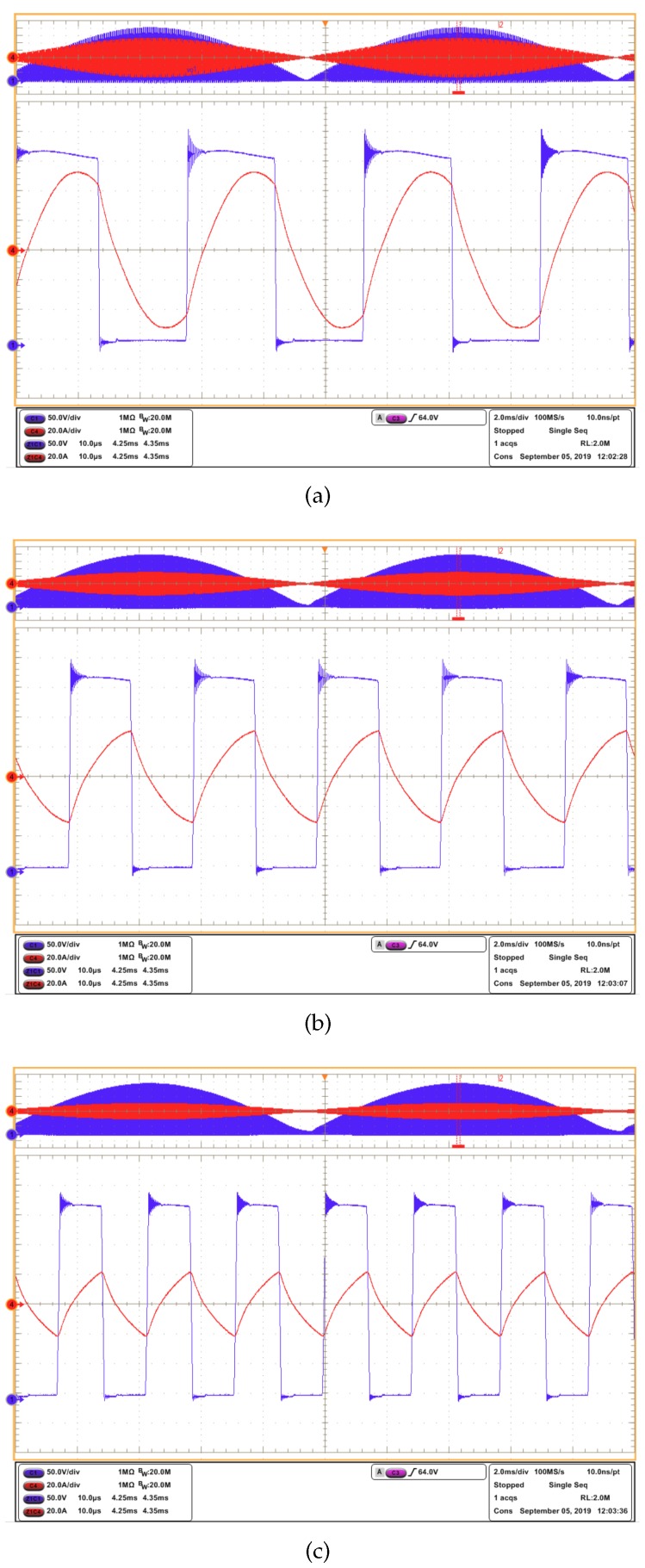
Experimental waveforms captured through the oscilloscope: Channel 1 (blue), $v_{O}$; Channel 4 (red), $i_{L}$, for switching frequencies of (**a**) 35 kHz, (**b**) 50 kHz, and (**c**) 70 kHz.

Figure 10 shows the inverter waveforms—voltage and current—captured with the oscilloscope at three switching frequencies in the range of interest. Table 4 shows the RMS voltage, RMS current, and power computed from the oscilloscope measurements, for the three switching frequencies.

**Figure 11:**
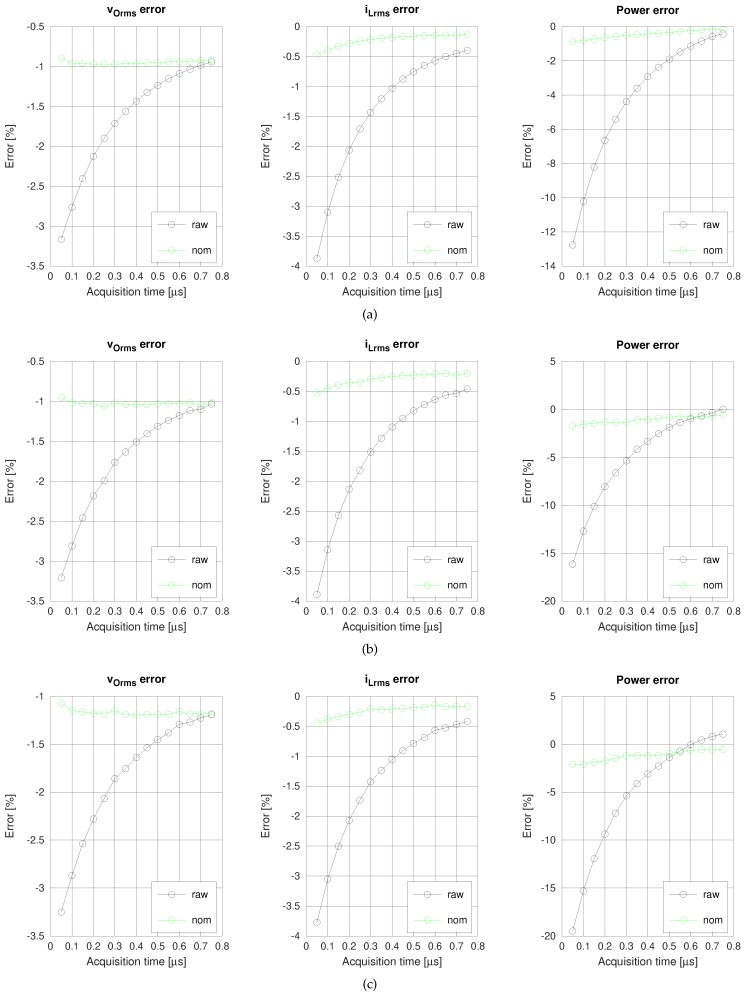
Voltage, current and power errors with raw data and correction method with nominal values for switching frequencies of (**a**) 35 kHz, (**b**) 50 kHz, and (**c**) 70 kHz.

Figure 11 compares the errors obtained from the raw-acquired data (raw) with the errors once corrected with nominal values (nom), for different acquisition times. The results for three switching frequencies are shown. Figure 11a corresponds to a low switching frequency (35 kHz), which delivers the highest power. Figure 11b corresponds to a medium switching frequency (50 kHz), and Figure 11c corresponds to a high switching frequency (70 kHz), which delivers the lowest power.

Without correction, voltage, current, and power errors decrease asymptotically as the acquisition time increases, provided the sampling frequency is enough to capture the harmonics of interest. If we had further increased the acquisition time, the errors would have grown due to the corresponding reduction of the sampling frequency. The maximum errors in absolute value are in the range of 3–4% for the voltage and current, and 13–20% for the power. Applying the correction method, even with nominal values, raises clearly the accuracy, with errors around 1% for the voltage and current, and less than 2% for the power.

**Figure 12:**
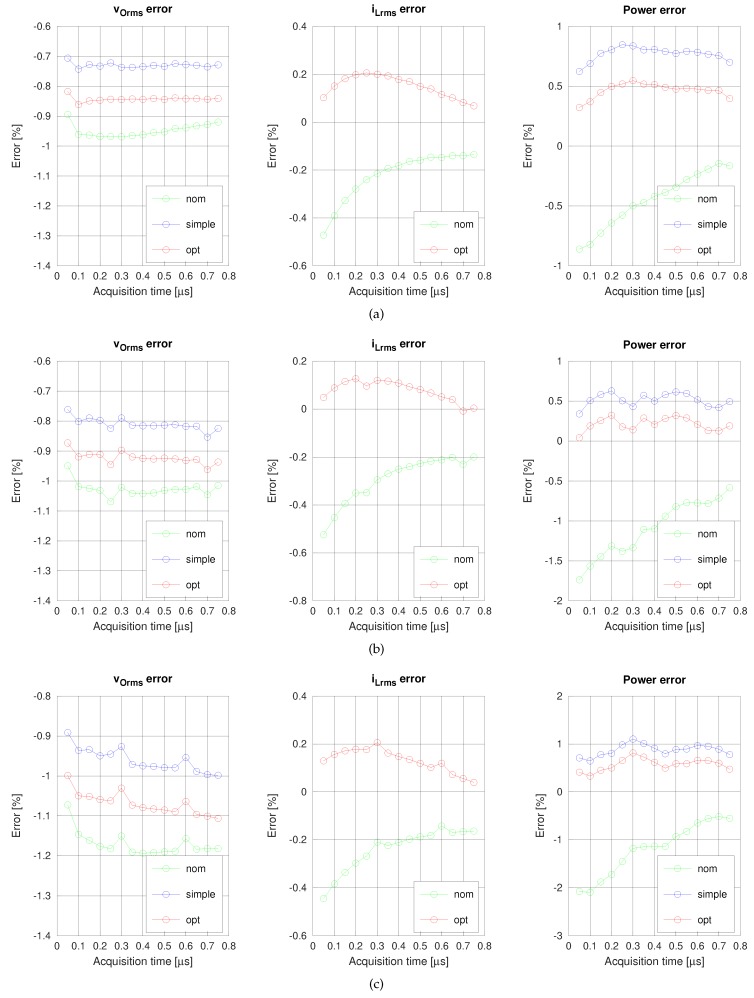
Nominal versus optimized voltage, current and power errors for switching frequencies of (**a**) 35 kHz, (**b**) 50 kHz, and (**c**) 70 kHz.

The accuracy can be further raised by using the parameters found by the minimization process. Figure 12 compares the errors when the measurements are corrected with nominal values (nom), the simple calibration process for voltage (simple), and the optimization-based method (opt). Results for the same switching frequencies as above are shown.

In all of the calibrated cases, the errors are decreased respect of the nominal case. More relevantly, the dependence of the error on the acquisition time has been reduced. The error curves for the simple and optimized cases are more horizontal than the nominal curves, especially for the current and power. Finally, it can be noticed that the worst behavior is for Figure 12c—the highest switching frequency case—surely due to the reduced range of the current to be measured in this case.

Table 5 summarizes the average and standard deviation of the power errors for the simple and optimized calibration methods. The fifteen acquisition time data points in the 50–750 ns range have been considered for computing the metrics. The simple calibration method achieves a worst-case average error of 0.87%, while the optimization-based method slightly reduces the worst-case average error (0.57%). The standard deviation is less dependent on the method, with a worst-case value of approximately 0.12%

**Figure 13:**
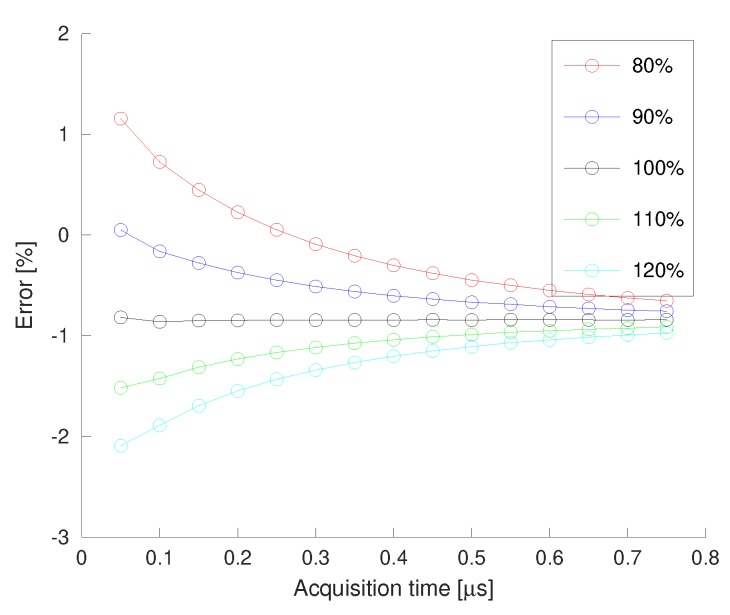
Voltage error for several $V_{SH0}$.

The value of $V_{SH0}$ must be selected carefully to achieve such a low error. The former experimental results have been obtained with a nominal $V_{SH0}=0.855V_{REF}/2$. Figure 13 shows the effect of changing $V_{SH0}$ on the RMS output voltage error. Five values of $V_{SH0}$ have been considered in the range 80–120% of its nominal value. Having a horizontal error curve with the acquisition error is a warranty of a good $V_{SH0}$ selection.

**Figure 14:**
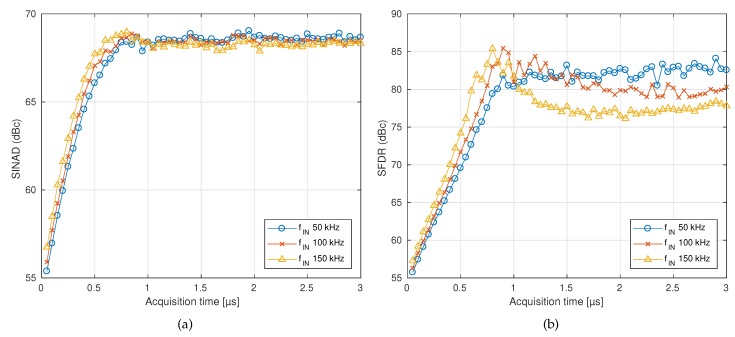
Spectral quality parameters of the ADC for different input frequencies and acquisition times. (**a**) signal-to-noise and distortion ratio (SINAD), (**b**) spurious-free dynamic range (SFDR).

Although this work is strictly focused on averaged parameters like power or RMS values, the ADCs are used under conditions their manufacturer does not recommend. The sampling process contains non-linear terms that degrade the performance of the conversion-chain when the 1/2 LSB constraint is ignored. This degradation can not be compensated with a linear method, so it could reduce the effective number of bits (ENOB) for instantaneous measurements. Some spectral quality parameters or frequency domain performance of the ADCs, for different times, have been measured. To do so, the circuit shown in Figure 4 was slightly modified: R1 and R2 were removed and their equivalent parallel resistance was placed between the positive terminal of $v_{o}$ and the input of the ADC. A waveform generator was connected to the input of the circuit and sinusoidal signals with the full-scale range (FSR) of the ADC at different frequencies were captured with different $t_{aq}$. For every case, 16k points were captured and the signal-to-noise and distortion ratio (SINAD) and spurious-free dynamic range (SFDR) were computed. The results of these two parameters are shown in Figure 14. It can be seen how the spectral quality of the ADC is reduced when low $t_{aq}$ values are imposed. Below 0.7 μs, the ENOB is reduced, but between 0.3–0.7 μs the reduction is less than 1 bit.

## 4. Discussion

A method to digitally compensate the errors due to the fact of not meeting the voltage settling constraint of 1/2 LSB, on SAR-ADCs without driver amplifiers, has been analyzed and experimentally tested. It is intended to reduce the cost, size, and power consumption of the conditioning front-end by eliminating the need for a driver amplifier. The compensation can be easily performed by the digital system in charge of the ADC’s control because it involves only one addition and one multiplication per sample.

The method has been experimentally applied to the output voltage and load current of a series resonant inverter operated in the switching frequency range of 35–70 kHz. Three relevant quantities have been computed from the acquired signals: the RMS output voltage, the RMS load current, and the power delivered to the load. All magnitudes have been computed over one half-cycle of the mains AC source.

The output voltage and load current have been captured by two SAR-ADCs driven with passive conditioning circuits. The SAR-ADCs are managed by a Zynq SoC that allows changing the acquisition time in integer multiples of the clock period. The voltage and current have been captured with acquisition times in the range 50–750 ns to investigate the error dependence on the acquisition time. The reference voltage and current to compare with have been captured with an oscilloscope in high-resolution mode and high sampling frequency.

Without any correction, voltage, current, and power errors decrease as the acquisition time increases. The maximum errors in absolute value are in the range of 3–4% for the voltage and current, and 13–20% for the power. The errors increase with the switching frequency.

A correction method based on the approximation of the ADC’s sample and hold circuit by a first-order RC network has been proposed to raise the accuracy. This method requires computing two correction parameters—$k_{1}$ and $k_{2}$—that depend on several circuit values ((10) and (11)). The parameters $k_{1}$ and $k_{2}$ can be computed from the nominal circuit values, obtaining the so-called nominal correction method. This method raises the accuracy, with resulting errors around 1% for the voltage and current, and less than 2% for the power.

The accuracy can be further increased by using the parameters found by a calibration process. Two possibilities have been proposed for the voltage: the simple method and the optimization-based method. Both are based on performing one first acquisition with a zero input signal. Only the optimization-based calibration method is available for the current.

Experimental results show that the errors for the calibrated cases are smaller than for the nominal case. More relevantly, the dependence of the error on the acquisition time is reduced. The error curves for the simple and optimized cases are more horizontal than the nominal curves, especially for the current and power. The simple calibration method achieves a worst-case average error of 0.87%, while the optimization-based method slightly reduces the worst-case average error (0.57%). The standard deviation is less dependent on the method, with a worst-case value of approximately 0.12%. Moreover, the calibrated methods can take into account variations due to manufacturing tolerance, temperature dependence, and aging of the components.

Therefore, depending on the accuracy constraints, we can select to apply the correction method with nominal values, or with the values found by the simple or optimization-based methods. Table 6 summarizes the applicability and complexity of the proposed correction methods.

Further work must be done to test the proposed acquisition and correction schemes to computing more parameters from the acquired signals. This paper has focused on computing some relevant averaged parameters—RMS values and power—but the operation and control of the power converter will require some other instantaneous or temporal parameters: peak current, switches’ turn on and turn off currents, or delay between current and voltage. From the results of the SINAD and the SFDR, it can be concluded that avoiding the driver amplifier in applications where high-accuracy instantaneous measurements are obtained from high-resolution ADCs may not be recommendable for low acquisition times. Nevertheless, the proposed method offers a good accuracy in the computation of averaged parameters even for very low acquisition times.

Future work will also address the online implementation of the optimization-based calibration methods. In this paper, a non-linear optimization algorithm has been implemented on a PC running Octave for proof-of-concept purpose. As this algorithm is intended to correct slow aging or temperature variations, it might be run a few times, typically once every several minutes. The ARM Cortex-A9 available on the Zynq SoC can be programmed to run a reduced version of this algorithm.

## Figures and Tables

**Table 1:** Component values for the output voltage conditioning circuit.

Name	Value	Type
R1	150 kΩ	1% metal film
R2	1.4 kΩ	1% metal film
C1	220 pF	1% NPO

**Table 2:** Component values for the load current conditioning circuit.

Name	Value	Type
CT	200:1	Current transformer
Rt	8.2 Ω	1% metal film
R3	1.3 kΩ	1% metal film
R4	1.3 kΩ	1% metal film
C2	470 pF	1% NPO

**Table 3:** Parameters obtained from the minimization process.

	$p_{1}$	$p_{2}$
Voltage	1.20938	1.08183
Current	1.33160	1.25660

**Table 4:** Voltage, current and power measured by the oscilloscope for different frequencies.

Freq (kHz)	$V_{\mathrm{Orms}}\left(V\right)$	$I_{\mathrm{Lrms}}\left(A\right)$	Power (W)
35	158.70	28.41	2309.49
50	159.18	15.23	934.92
70	158.32	10.39	548.44

**Table 5:** Average and standard deviation of the power errors.

Freq (kHz)	Average (%)		Standard Deviation (%)	
	Simple	opt	Simple	opt
35	0.7698	0.4655	0.0589	0.0605
50	0.5134	0.2100	0.0832	0.0814
70	0.8740	0.5708	0.1221	0.1269

**Table 6:** Qualitative comparative of the correction methods.

	Nominal	Simple	Optimization
Applicable to voltage	Yes	Yes	Yes
Applicable to current	Yes	No	Yes
Complexity	Low	Low	High
